# Adherence and acceptability of multiple micronutrient supplementation during pregnancy: Study protocol for a cluster-randomized non-inferiority trial in Cambodia

**DOI:** 10.1186/s13063-023-07891-z

**Published:** 2024-04-29

**Authors:** Mai-Anh Hoang, Hou Kroeun, Rolf Klemm, Aman Sen Gupta, Ngik Rem, Sokchea Meng, Sophonneary Prak, Kim Rattana, Mary Chea, Crystal D. Karakochuk, Cassandra Sauer, Ashutosh Mishra, Diwakar Mohan, Meredith Jackson de-Graffenried

**Affiliations:** 1 Helen Keller Intl, Asia Pacific, Manila, Philippines; 2 Helen Keller Intl, Cambodia Country Office, Phnom Penh, Cambodia; 3 Ministry of Health, Phnom Penh, Cambodia; 4 Division of Nutrition, Johns Hopkins Bloomberg School of Public Health, Baltimore, MD USA; 5 Food, Nutrition and Health, The University of British Columbia, Vancouver, British Colombia Canada; 6 Vitamin Angel Alliance, Goleta, CA USA; 7 Department of International Health, Johns Hopkins Bloomberg School of Public Health, Baltimore, MD USA; 8 Helen Keller Intl, North America, New York, United States

**Keywords:** Acceptability, Adherence, Antenatal, Cambodia, Iron and folic acid, Multiple micronutrients, Pregnancy, Supplementation, Non-inferiority trial

## Abstract

**Background:**

Iron and folic acid (IFA) supplements are currently provided to Cambodian women during pregnancy. However, recent research has found benefits of a multiple micronutrient supplement (MMS) over just IFA alone on several outcomes of perinatal and infant health. The Ministry of Health in Cambodia has proposed a transition from IFA to MMS but to effectively guide this transition requires implementation research on the acceptability and adherence to MMS (over IFA).

**Methods:**

This non-inferiority trial aims to assess the adherence and acceptability of IFA (60 mg elemental iron and 400 μg folic acid) compared to MMS (standard UNIMMAP formulation including 15 micronutrients) during antenatal care in Cambodia. A prospective cohort of 1545 pregnant women will be assigned to one of three trial arms: (1) IFA for 90 days [IFA-90]; (2) MMS for 180 days with two distributions of 90-count tablet bottles [MMS-90]; or (3) MMS for 180 days with one 180-count tablet bottle [MMS-180]. Each arm will enroll 515 women across 48 health centers (clusters) in Kampong Thom Province in Cambodia. The primary outcome is the non-inferiority of adherence rates of MMS-180 compared to IFA-90, as assessed by tablet counts. Mixed-effects logistic and linear regression models will be used to estimate the difference in the adherence rate between the two groups, with an ‘a priori’ determined non-inferiority margin of 15%. Acceptability of MMS and IFA will be measured using a quantitative survey conducted with enrolled pregnant women at 30-day, 90-day, and 180-day time-points.

**Discussion:**

Findings from this study will guide an effective and feasible MMS scale-up strategy for Cambodia. Additionally, the findings will be shared globally with other stakeholders planning to scale up MMS in other countries.

**Trial registration:**

NCT05867836 (ClinicalTrials.gov, registered May 18, 2023).

**Supplementary Information:**

The online version contains supplementary material available at 10.1186/s13063-023-07891-z.

## Administrative information

**Table:** 

***Title {1}***	Adherence and acceptability of multiple micronutrient supplementation among pregnant women in Cambodia: A cluster-randomized non-inferiority trial
***Trial registration {2a and 2b}***	NCT05867836
***Protocol version {3}***	Version 4_May 22, 2023
***Funding {4}***	This study is funded through Vitamin Angels Alliance, a global non-profit organization with headquarters in Santa Barbara, California, USA.
***Author details {5a}***	**Mai-Anh Hoang** (first author), Helen Keller Intl (Helen Keller) Role: acquired funding; conceptualized protocol design and methodology; developed study tools; standard operating procedures; provided technical oversight of training data collectors and study implementation; and writing, review, and editing of study manuscript. **Meredith Jackson de Graffenried** (last author), Helen Keller Intl. Role: conceptualization of protocol design and methodology, developed study tools. **Hou Kroeun** (second author and corresponding author), Helen Keller Intl. Role: Acquired funding, support project administration and supervision, validation of findings; writing-review and editing **Rolf Klemm** (co-author), Helen Keller Intl. Role: Acquired funding, conceptualized protocol design and methodology; interpretation of data; writing review and editing. **Aman Sen Gupta** (co-author), Helen Keller Intl. Role: conceptualized protocol design and methodology, provided technical oversight of training data collectors; data curation, project supervision; data management; data validation; data analysis; data interpretation, writing-review and editing. **Rem Ngik** (co-author), Helen Keller Intl. Role: conceptualized protocol design and methodology, provided technical oversight of training data collectors; data curation; project supervision; data management; data interpretation; writing-review and editing. **Meng Sokchea** (co-author), Helen Keller Intl. Role: provided technical oversight of training data collectors; data curation, project supervision; data management; data validation; interpretation of findings. **H.E. Dr. Prak Sophonneary,** (co-author), Cambodia, Ministry of Health. Role: supervision; interpretation of data; writing-review editing. **Kim Rattana,** (co-author), Cambodia, National Maternal and Child Health Center. Role: supervision; interpretation of data; writing-review editing. **Chea Mary,** (co-author), Cambodia, National Nutrition Program. Role: supervision; interpretation of data; writing-review editing. **Crystal D Karakochuk** (co-author), The University of British Columbia. Role: Project supervision, validation, data analysis, interpretation of data; drafting original study report/manuscript. **Cassandra Sauer** (co-author), The University of British Columbia. Role: Project supervision; contributed to study tools and standard operating procedures, and implementation guidelines; data analysis; interpretation of data; drafting original study report/manuscript. **Ashutosh Mishra** (co-author), Vitamin Angels Alliance. Role: Acquired funding/funder, conceptualized protocol design; project supervision; interpretation of data.
***Name and contact information for the trial sponsor {5a}***	Crystal D. Karakochuk PhD, RD The University of British Columbia 216–2205 East Mall Vancouver BC | V6T 1Z4 Canada Phone + 1 60 8220 421
***Role of sponsor {5c}***	Support for quality implementation of research protocol; data analysis; data interpretation; and original writing and reporting of research results.
***Composition, roles and responsibilities of steering committee, data management team, and other groups overseeing the trial {5d}***	The MMS Steering Committee members are responsible for providing technical review and inputs to the design of the study protocol; to help coordinate with relevant subnational stakeholders for smooth implementation of the trial; observe study implementation and provide feedback and recommendations to the study team; provide technical interpretation of study findings; and, help facilitate the sharing of project findings with relevant stakeholders.

## Introduction

### Background and rationale {6a}

Antenatal multiple micronutrient supplementation (MMS) is a potentially cost-effective, scalable approach that can help address the persistent challenge of maternal undernutrition, reduce the incidence of low birth weight (LBW), decrease the number of small-for-gestational age births, and potentially reduce the prevalence of preterm birth. The individual patient data meta-analysis demonstrated that MMS decreased mortality for female neonates and provided grater reductions in the risk of LBW and preterm briths for infants born to undernourished and anemic women. [1] The 2019 *Cochrane Review* and meta-analysis compared the effects of MMS to iron folic acid (IFA) supplementation and found that MMS was associated with reduced small-for-gestational age births [2]. Despite the potential benefits, the adoption of the guidelines on antenatal MMS into national policies has been limited, partly because the current WHO guidelines recommend MMS integration in antenatal care (ANC) services, in the context of rigorous research [3, 4]. To ensure successful nationwide adoption of MMS necessitates assessments and robust planning for optimal integration into existing antenatal care platforms. 

Helen Keller Intl (Helen Keller), with support from Vitamin Angel Alliance, conducted a landscape analysis of antenatal MMS in Cambodia to engage stakeholders who were deemed influential to accelerating MMS uptake and use in the country. During a consultation workshop held in September 2021, stakeholders concluded that it is an appropriate time to consider the transitioning from IFA to MMS. Participants at the consultation workshop also identified key research priorities related to the operational and implementation aspects of the transition process. One of the priorities is to understand pregnant women's acceptability and adherence of MMS, including factors that could influence perception of the prenatal supplement such as its packaging, branding and marketing. Subsequently, a multi-stakeholder Steering Committee comprising of technical and government representatives from various disciplines was formed to oversee and support this transition. The committee developed a terms of reference, which were endorsed by the Cambodia Ministry of Health (MoH).

### Rationale for the study

The strategy to transition from IFA to MMS as a new intervention in routine ANC services requires an assessment of the acceptability and feasibility among end users before implementation. Assessing the acceptability of MMS by pregnant women will provide insights into implementation barriers and contribute to a cost-effective and feasible scale-up strategy for MMS. Implementation strategies should consider various factors that influence acceptability and adherence, including MMS product availability and affordability, and antenatal health-seeking behaviors. It is also crucial to determine the non-inferiority of adherence when introducing a new supplement. Given the lack of studies and knowledge on MMS in Cambodia, the Cambodia MoH, Helen Keller, The University of British Columbia, and Vitamin Angel Alliance propose implementation research to identify factors influencing adherence to antenatal supplementation to effectively inform an MMS strategy within ANC service delivery.

UNIMMAP MMS (United Nations International Multiple Micronutrient Antenatal Preparation Multiple Micronutrient Supplements) is not a new product; however the majority of pregnant women and health providers in Cambodia are not familiar with this prenatal supplement. As MMS is intended to replace IFA, it is essential to introduce and transition to MMS carefully to ensure optimal acceptability and adherence. Without such measures, improving maternal nutrition may remain a challenge. As suggested by others transitioning to MMS requires a formative research phase to inform programming and service delivery [5]. This phase should involve testing product acceptability, labeling, packaging, health worker capacity building strategies, and messaging to promote daily use among Cambodian pregnant women. Additionally, it is necessary to explore concerns expressed by host-country stakeholders, including the impact on ANC attendance (ensuring at least four visits) if MMS is provided in a large quantity (i.e., 90 or 180 tablets) to pregnant women.

The primary knowledge gap to be addressed is the level of adherence to the recommended 180 tablets of MMS during pregnancy. Non-inferiority of adherence rates (based on pill counts) to MMS-180 compared to IFA-90, using a non-inferiority margin of 15%. To assess individual adherence, the total number of tablets consumed, estimated based on actual tablet counts will be calculated for each woman and divided by the number of tablets the woman was eligible to take at the time of the survey. A secondary analysis will be to assess the non-inferiority of adherence rates of IFA-90 compared to MMS-90 as well as MMS-180 compared to MMS-90, all using a non-inferiority margin of 15%.

### Objectives {7}

The aim of this study is to assess the acceptability and adherence to MMS as compared to IFA and to explore factors that may influence optimal MMS consumption, defined as 180 tablets during pregnancy. Findings will guide the Cambodia MOH and the MMS Steering Committee in developing an effective strategy for distributing 180 tablets of MMS as part of routine ANC services.

This study has the following objectives:

1. Assess non-inferiority of MMS compared to IFA supplementation.
2. Assess adherence to recommended IFA and MMS.
3. Assess acceptability of IFA and MMS.
4. Assess impact of tablet quantity provided at one time on ANC attendance.
5. Evaluate factors that influence adherence behavior.

### Trial design {8}

This study uses a cluster-randomized non-inferiority control trial design. The trial uses tablet counts to assess adherence, as well as a survey to evaluate acceptability. The trial will be conducted among a cohort of pregnant women assigned to one of three trial arms (*described below in section intervention description*). Data collection will take place over a period of 12 months.

## Methods: participation, interventions, and outcomes

### Study setting {9}

The study will be conducted in Kampong Thom Province, located in the central region of Cambodia. Kampong Thom Province is a semi-rural area with peri-urban populations and represents a suitable setting for piloting and studying the transition to MMS. The study will be conducted in three health operational districts (ODs) within Kampong Thom Province: Kampong Thom, Baray-Santuk, and Staung. These districts have a total population of approximately 780,000, with an estimated 17,429 pregnant women annually. The study will recruit pregnant women from 48 out of the 55 health centers in these districts.

### Eligibility criteria {10}

To be eligible for the study, participants must meet the following criteria:

1. Age 18–45 years
2. Currently pregnant with gestational age of less than 14 weeks
3. Low-risk pregnancy which includes being pregnant with a single pregnancy, vertex pregnancy, and the absence of any other medical or surgical conditions
4. Resides in Kampong Thom and not planning to move away within 6 months
5. Not participating in any nutrition programs beyond the normal care provided through government facilities
6. Agree to have data collectors conduct monthly home visits to collect data and conduct tablet counting

### Who will take informed consent? {26a}

If the pregnant women meet the eligibility criteria and indicate a willingness to enroll, the midwife will share the woman’s contact information with the research field officer assigned to that health center, who then obtains written informed consent.

### Additional consent provisions for collection and use of participant data and biological specimens {26b}

Not applicable—No provisions are required for collection and use of participant data and no biological specimens will be collected in this study.

## Interventions

### Explanation for choice of comparators {6b}

WHO recommends that all pregnant women receive a standard dose of 30–60 mg iron and 400 μg folic acid as early as possible during pregnancy [6]. Ideally, women should receive IFA no later than the first trimester of pregnancy and take 180 tablets until delivery. In the current standard of care in Cambodia, pregnant women receive a total of 90 tablets of IFA supplements through ANC. This distribution is typically organized as follows: 60 tablets are provided during the first ANC visit, and an additional 30 tablets are given during the second ANC visit. Cambodia recommends four ANC visits during pregnancy, which also includes essential nutritional counseling and information about the importance of taking prenatal supplements.

The most recent Cambodia Demographic Health Survey (CDHS 2021–2022) does indicate that 86% of women had four or more ANC visits for their most recent live birth or stillbirth, and 98% of women took iron-containing supplements during their most recent pregnancy. Despite these figures, there is a data gap in knowing the actual adherence rates to the recommended 180 tablets of IFA, especially considering the government’s current distribution of only 90 IFA tablets. This underscores the importance of our study, as we seek to assess adherence and acceptability to 180 tablets of MMS as a potential alternative to IFA.

To date there are no published studies or documents from Cambodia on pregnant women’s acceptability of MMS or adherence to a 180-dosage regimen. A 2011 study in two provinces in Cambodia found access to ANC, the number of supplements provided, and ANC attendance were the strongest determinants for adherence to IFA supplementation [7]. Another assessement in Cambodia found the primary reasons for not adhering to 180 tablets of IFA during pregnancy were starting ANC after the first trimester and not attending all recommended four ANC visits [8]. In Nepal, Rai et al., found knowledge about preventable conditions and benefits, perceived barriers, social support, and perceived severity of not taking the supplement predicted IFA adherence [9] while Kulkarni et al. also found forgetting to take the supplements was a barrier [10]. In a study from Vietnam [11], determinants of adherence to either folic acid, IFA, or MMS were socioeconomic status, ethnicity, occupation, and parity, as well as increased contact with health workers. Acceptability studies which included MMS have identified organoleptic properties, perceived benefits, and fears or perceived negative effects as influential factors on acceptance and utilization [7, 12].

Assessment of the acceptability and feasibility of a new intervention should be done with end users prior to implementation, especially when the strategy requires possible changes in care processes. Determining the acceptability of MMS with pregnant women will enhance understanding of barriers to implementation and help to develop an MMS scale-up strategy that is feasible and cost-effective. Determining non-inferiority of adherence is also critical when introducing a new supplement.

In conclusion, the rationale for our study lies in the introduction of 180-tablets of MMS within ANC. To ensure successful implementation and scale-up of MMS, implementation research has been widely accepted as a necessary step [7]. This phase will involve testing product acceptability and adherence. Other planned studies include assessing enablers and barriers to MMS acceptability and adherence, product packaging and labeling, product marketing, health worker capacity building strategies, and strategic behavioral change communication strategies to promote daily use among Cambodian pregnant women. Furthermore, we need to address concerns expressed by host-country stakeholders regarding the potential impact on ANC attendance if MMS is provided in a larger quantity (i.e., 90 or 180 tablet bottles). Therefore, our study aims to bridge these knowledge gaps and inform the transition and scale-up of MMS in Cambodia’s ANC service delivery.

### Intervention description {11a}

The trial will include three intervention arms:

1. IFA-90: Women in this arm will receive 60 tablets of IFA at the first ANC visit (ANC1) and 30 tablets of IFA at the second ANC visit (ANC2), as per current Cambodia's MoH guidelines
2. MMS-90: Women in this arm will receive a total of 180 tablets of MMS, with 90 tablets distributed at ANC1 and 90 tablets distributed at ANC2
3. MMS-180: Women in this arm will receive 180 tablets of MMS distributed at ANC1

The IFA tablet contains 60 mg elemental iron and 400 μg folic acid, while the MMS formulation was based on the UNIMMAP formulation [13] and contains 15 micronutrients: 30 mg elemental iron, 400 μg folic acid, 800 μg vitamin A, 200 IU vitamin D, 10 μg vitamin E, 70 mg vitamin C, 1.4 mg thiamin, 1.4 mg riboflavin, 18 mg niacin, 1.9 mg vitamin B6, 2.6 μg vitamin B12, 2 mg copper, 150 μg iodine, 65 μg selenium, and 15 mg zinc. The MMS tablets are available in either 90-tablet or 180-tablet bottles. Health centers (clusters) will be randomly assigned to one of the three arms.

### Criteria for discontinuing or modifying allocated interventions {11b}

Pregnant women enrolled in the trial who experience any reported adverse reactions in any of the trial arms will be referred to their midwife or doctor for guidance on whether to continue in the study or to withdraw. Participants are free to withdraw at anytime for any reason without consequence.

### Strategies to improve adherence to interventions {11c}

ANC and research staff will provide standard of care strategies to improve adherence to prenatal vitamins, such as counseling on the benefits of prenatal vitamins and how best to manage side-effects. The study aims to assess adherence of the interventions without influencing outcomes.

### Relevant concomitant care permitted or prohibited during the trial {11d}

In the IFA-90 arm, pregnant women identified with anemia (Hb < 11 g/dL) during pregnancy will receive the Cambodia MoH’s standard of care, which includes provision of two IFA tablets per day and monitoring to assess if hemoglobin concentrations increase. Anemia is routinely tested for using the Hemocue at ANC1.

For the MMS-90 and MMS-180 trial arms, pregnant women with mild or moderate anemia (Hb 7.9– < 11.0 g/dL) will be referred to their midwife or doctor for guidance on the care and/or treatment of anemia during pregnancy.

### Provision for post-trial care {30}

Post-trial care will follow the Cambodia MoH’s standard of care protocols for all enrolled participants.

### Outcomes {12}

#### Primary Outcome

The primary outcome is the non-inferiority of adherence rates (based on pill counts) of MMS-180 (180 days) compared to IFA-90 (90 days), using a non-inferiority margin of 15%. Adherence will be calculated based on the number of tablets consumed, divided by the total number of tablets the woman was eligible to take at the time of the final study visit.

#### Secondary Adherence Outcomes

The non-inferiority of adherence rates, (based on pill counts) of MMS-180 (180 days) compared to MMS-90 (180 days), using a non-inferiority margin of 15%.

The non-inferiority of adherence rates, (based on pill counts) of MMS-90 (90 days) compared to IFA-90 (90 days), using a non-inferiority margin of 15%.

#### Other Acceptability Outcomes

The overall acceptability of MMS as compared to IFA, assessed at the end-point of the trial (90 days for IFA-90 and MMS-90 and 180 days for MMS-180).

##### Adherence (%):

The non-inferiority of adherence rates, (based on tablet counts) of MMS-180 (180 days) compared to MMS-90 (180 days), using a non-inferiority margin of 15%. M*MS-1890 compared to IFA-90 at the trial's end-point will be measured. in terms of adherence rates. The non-inferiority of adherence rates, (based on pill counts) of MMS-90 (1890 days) compared to IFA-90 (90 days)MMS-180 to MMS-90 in terms of adherence rates. Other Acceptability Outcomes: The overall.*

### Participant timeline {13}

**Fig. 1:**
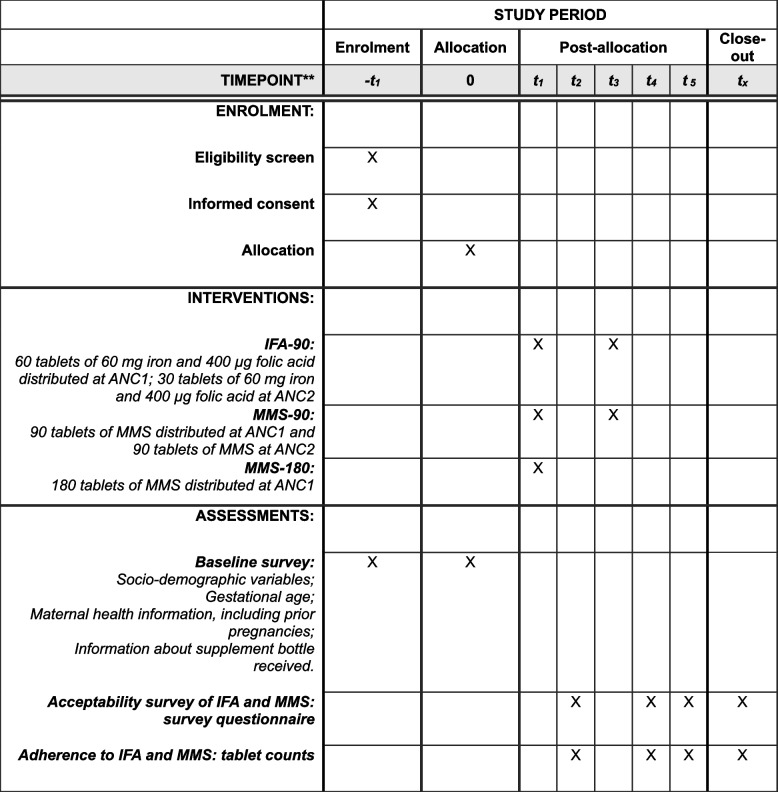
Schedule of enrolment, interventions, and assessments

Figure 1 outlines the schedule of enrollment, interventions, and assessments.

***t***_***1***_-ANC 1: Distribution of prenatal supplements for all three trial arms.

***t***_***2−***_ 30-day house visit: Acceptability survey questionnaire and adherence tablet count for IFA-90, MMS-90, and MMS-180.

***t***_***3***_-ANC 2: Distribution of prenatal supplements for IFA-90 and MMS-90 groups.

***t***_***4***_–90-day house visit: Acceptability survey questionnaire and tablet count for IFA-90, MMS-90, and MMS-180.

***t***_***5***_–180-day house visit: Acceptability survey questionnaire, tablet count for MMS-90 and MMS-180 groups.

### Sample size {14}

A sample of 1545 pregnant women is required for the statistical power determination for the adherence non-inferiority tests. Sample size estimations were calculated using adherence as both a binary and a continuous outcome. The more conservative sample size calculation for adherence as a binary outcome was chosen to ensure we were powered for both outcomes. The sample size was determined using a design effect of 2, twice as large as would be expected with simple random sampling. This accounts for the clustering within health centers (across 48 clusters) and the testing of three different arms. As the study arms are all within the same province and within three similar operational districts, large differences within clusters are not expected. Therefore, a design effect of 2 was deemede appropriate.

In addition, the sample size was calculated considering a 2-sided test, 90% power, an alpha of .025 for a one-sided confidence interval (CI), and a non-inferiority parameter of 15%. This sample size will allow for the detection of a significant difference in intake between the arms equal to 2% prevalence difference between arms (76% vs 74%) and a non-inferiority limit was determined ‘a priori’ as 15%. To compensate for a potential 10% loss to follow-up, a total of 515 women per arm will be recruited. Thus, the total sample size of pregnant women enrolled in the non-inferiority trial is 1545.

### Recruitment {15}

The trial will randomly assign 48 government run health centers (clusters) from three Operational Districts to one of the three arms. Each arm will distribute a different supplement: IFA-90, MMS-90, or MMS-180. The MMS will only be given to enrolled trial participants.Pregnant women will be enrolled on a rolling basis in each health center. The enrollment process is anticipated to be completed within 3 months. Pregnant women will be enrolled on a rolling basis in each health center. The enrollment process is anticipated to be completed within 3 months.

Incentives for participant enrollment are not provided, but several strategies will be employed for ensuring sufficient cluster and participant enrollment. An orientation session for ANC providers (maternal and child health chiefs, midwives, and nurses) will be conducted to ensure a clear understanding of the study interventions and enrollment criteria. This session aims to equip health facility staff with the necessary knowledge and skills for effective enrollment of eligible participants.

Training sessions conducted by the research team to all health facility staff will emphasize the importance of prenatal vitamins in promoting a healthy pregnancy. Staff we will be encouraged to communicate this vital information to all participants across all study arms. This aims to raise awareness among pregnant women about the benefits of the study interventions, regardless of their assigned arm, and to underscore the significance of their participation.

## Assignment of interventions: allocation

### Sequence generation {16a}

Excel software generated a random allocation sequence by assigning 48 health centers to one of the three arms. There were 56 health centers in three working operational districts Kampong Thom [23], Baray Santuk [22], and Staung [11]. Data on 2022 annual pregnancy rates in each health facility were taken from the operational districts. Using the average number of pregnant women at each center, the research team calculated the number of enrollments needed per month per arm to achieve the desired sample size within 3 months. This calculation determined 48 health centers were sufficient. The 48 centers were proportionately divided across the three districts. Within each district, health centers were identified using probability proportion to size (PPS) to ensure equal selection probability regardless of center size. To avoid bias, each health centers was randomly assigned to a group (0=IFA, 1=MMS-180, 2=MMS-90).

To ensure sequence generation will be unbiased, sequence generation will be conducted by an external analyst residing outside of the country who will have no information on the clusters to bias the sequence allocation (e.g., no knowledge of the geographical nature or organization of the health facilities and operational districts in Cambodia).

### Concealment mechanism {16b}

 Staff at each health facility will enroll participants based on the intervention arm assigned to that specific facility. There will be no concealment of the intervention groups because each health facility was randomly allocated to receive one of the three interventions.

The rationale for this approach is two-fold: (1) Logistical challenges: The distribution and management of MMS is anticipated to pose logistical challenges. Randomly assigning individuals to the treatment arms will require maintaining appropriate levels of MMS supply at all 48 health facilities. Given the complexities involved in ensuring a consistent supply of MMS, the decision was made to allocate interventions at the health facility level, which allowed for more manageable logistics. (2) Feedback on user experience: The MMS Steering Committee, consisting of MOH colleagues, recognized the importance of closely monitoring the introduction of MMS. By allocating interventions at the health facility level, they will retain the ability to visit these facilities regularly. This will enable the MOH to gather feedback from health provides regarding patient experiences and user feedback, ensuring that any issues or challenges could be addressed promptly.

### Implementation {16c}

Midwives will screen all pregnant women for eligibility and their willingness to enroll in the study. If a woman is eligible and willing to enroll, the midwife will share her contact information with the research coordinator assigned to that health center. The research officer will contact the woman within 24 h and arrange a meeting time at her home to obtain written informed consent.

Each of the 48 health facilities will aim to enroll between 25 and 45 pregnant women each, until each study arm reaches 515 participants. To ensure balanced enrollment across facilities, the research coordinator for each operational district will assess progress weekly. This assessment will prevent any single facility from surpassing the maximum enrollment of 45 women.

## Assignment of interventions: allocation

### Who will be blinded {17a}

Study participants, the investigators, outcome assessors, and data collectors will not be blinded to their treatment arm and will be informed whether they are assigned to receive IFA or MMS. The rationale for this approach is as follows:

1. Differential data collection points: One of the primary reasons for not implementing blinding in our study is the difference in data collection points for each study arm. The study arms will have varying schedules for data collection based on the specific intervention they were receiving. In the IFA-90 Study Arm, pregnant women will be followed up for 90 days post-enrollment. This duration aligns with the standard of care for pregnant women in Cambodia, who typically receive 90 IFA pills during pregnancy. In the MMS-90 and MMS-180 study arms—the World Health Organization recommends MMS supplementation for at least 180 days during pregnancy—additional data collection points will be needed for these groups, including an additional endline survey and pill count at 180 days post-enrollment or until conception occurred.
2. Consideration for tablets consumed: Given the variation in the duration of supplement consumption (90 days for IFA-90 vs. approximately 180 days for MMS-90 and MMS-180), data analysis will need to account for the number of tablets consumed relative to the days available for tablet consumption. Consequently, it was important to identify and differentiate women in the two MMS study arms, as they had a longer period to consume MMS tablets.

### Procedure for unblinding if needed {17b}

Not applicable.

## Data collection and management

### Data collection method {18a}

All quantitative data will be electronically captured on Android devices using Open Data Kit (ODK) Collect. The questionnaires will be initially developed in English and subsequently translated into Khmer, the local language. To ensure accuracy, they will be back-translated into English before the data collection process commences. Furthermore, all questionnaires will go through pretesting prior to being transferred into the ODK platform.

Throughout the study, a diligent record of all women who will be screened but not enrolled will be kept in ONA [14]. Participants who provide verbal informed consent at the health center will be given up to a maximum of 2 weeks to complete their written consent with the enumerator at the participants’ home.

Adherence monitoring will take place at the participants’ homes. Tablet counts will be conducted at the 30-day and 90-day time-points for the IFA-90 group. The MMS-90 and MMS-180 group will have tablet counts at the 90-day and 180-day time points.

To evaluate the acceptability of MMS and IFA over time, a longitudinal survey assessing organoleptic properties with a 5-point Likert scale will be administered to participants in the three study arms at 30 days and 90 days post-enrollment. Both MMS-90 and MMS-180 groups will be administered the survey again at 180 days post-enrollment.

### Plans to promote participant retention and complete follow-up {18b}

There are no plans to promote participant retention and complete follow-up. However, as a token of appreciation for participating in the study and agreeing to the house visits and interviews, participants will be provided a sarong at the first visit and a towel at the last visit. Participants in MMS-90 and MMS-180 will also receive a bar of soap at the 90-day visit.

### Data management {19}

Survey forms will be created in ONA and downloaded onto ODK Collect for enumerators to administer the surveys with participants. Enumerators will be obligated to verify and submit all finalized surveys back to ONA daily. Once the data is successfully submitted to the server, data cleaning will be conducted, including data quality checks for outliers, missing values, and patterns in all variables to ensure data quality and reliability. Administrative control over form and data uploading and downloading will be maintained by the study’s data collection team.

### Confidentiality {27}

All participant data will be anonymized, and participants will be assigned a unique ID number. Password protection will provide an additional safety caveat for confidentiality. Participant data will be collected through the digital data collection platform Ona and managed in SPSS (which are password-protected secure sites under management by Helen Keller International standard of practice). All laptops which have access to this site are password protected and the data in the site is also password protected. All unattended devices will be in kept in a locked room with restricted access. Only authorized individuals will have access to keys or swipe cards that have access to devices. We will be sure to take all security measures to prevent our devices from being seen, or stolen. Once all the data is collected, the principal investigator will receive the final data file. This will be transferred from Helen Keller International in Cambodia to the University of British Columbia via UBC Workspace (a safe, university-hosted cloud-based sharing service).

### Plans for collection, laboratory evaluation and storage of biological specimens for genetic or molecular analysis in this trial/future use {33}

No biological specimens will be collected in this trial.

## Statistical methods

### Statistical methods for primary and secondary outcomes {20a}

#### Primary and secondary outcomes

The primary comparison of interest is the non-inferiority of MMS-180 compared to IFA-90 in terms of adherence rates. We will assess adherence as both a continuous outcome (assessing the mean [95% CI] difference in adherence rates across the MMS and IFA groups using mixed-effects linear regression models) and a binary outcome (assessing the proportion of individuals who were ‘successfully’ adherent above a threshold of 76% adherence across the groups using mixed-effects logistic regression models). We will adjust for health centers (clusters) and confounding variables using Stata software. We have defined a non-inferiority margin of 15% adherence, and this comparison will be the focus of our primary analysis. The analysis will follow an intention to treat principle, meaning that all participants will be analyzed according to their originally allocated intervention group, regardless of deviations including loss to follow-up, withdrawal, or non-compliance.

The selection of the 15% margin of non-inferiority was based on several factors, including clinical relevance, existing literature, and expert consultation. It was also supported by consultations with experts in maternal and child health and nutrition. A margin of 15% was considered a reasonable threshold for non-inferiority, given that it allows for a clinically relevant difference in adherence rates between the intervention arms while maintaining the overall effectiveness of the interventions.

In addition to the primary analysis, we will conduct secondary analyses to compare:

- MMS-90 to IFA-90 in terms of adherence rates.
- MMS-180 to MMS-90 in terms of adherence rates.

Adherence will be calculated by dividing the total number of tablets consumed (based on tablet counts) by the number of tablets the woman was eligible to take during the survey period. The formula for calculating endpoint adherence is as follows:

- Tablets Consumed: Tablets received − Tablets remaining at final study visit.
- Days available to consume tablets: ANC 1 Date − Final Study Visit Date.
- Endpoint Adherence (%): (Tablets Consumed / Days available to consume tablets) × 100.

We will present and interpret the adherence outcomes using a 95% CI in relation to the ‘a priori’ determined 15% non-inferiority margin. The below figure are potential outcomes that could be seen in our trial and how they would be interpreted. Each of the error bars indicates a 2-sided 95% CI and the black squares are the point estimates. Example A shows that MMS adherence would be non-inferior IFA adherence, since the CI lies fully to the right of the non-inferiority margin [15]. Example B indicates that MMS adherence is inferior to IFA adherence, based on the CI being to the left of the non-inferiority margin. 

**Fig. 2:**
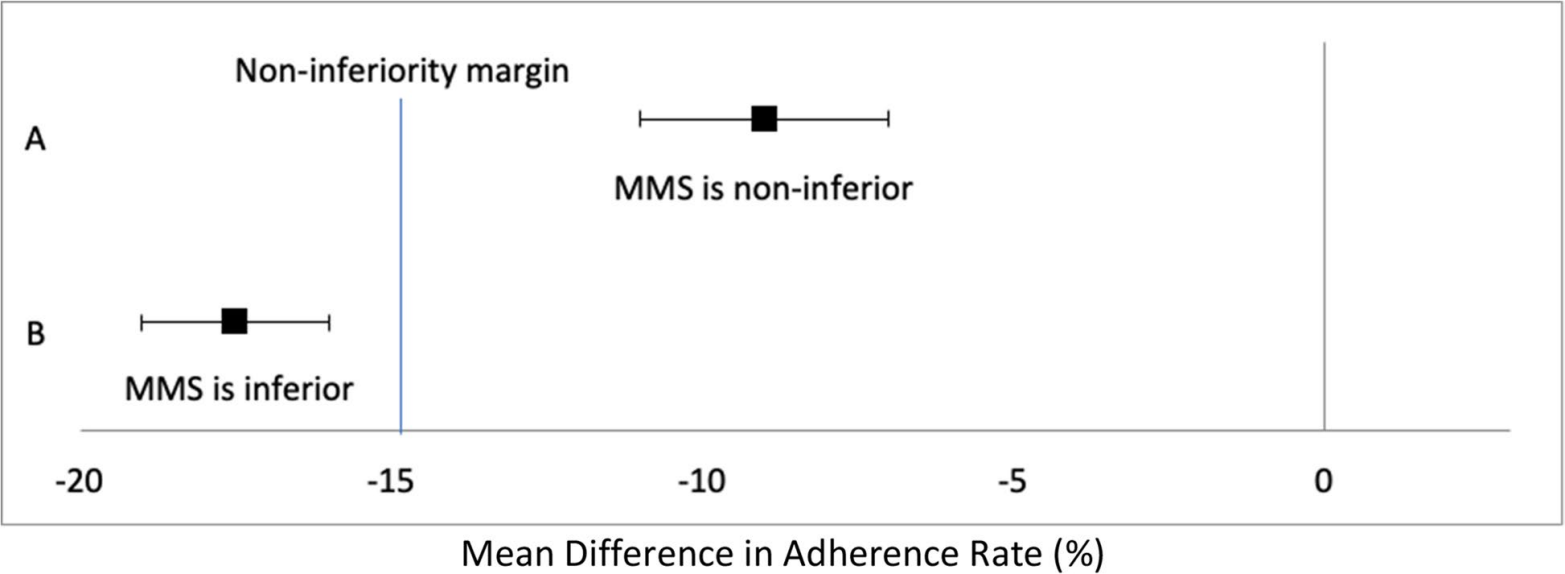
Possible scenarios for adherence outcomes in the non-inferiority trial

The primary outcome of non-inferiority of MMS-180 compared to IFA-90 will be assessed using Fig. 2 below, as well as the secondary analysis of MMS-90 to IFA-90 and MMS-180 to IFA-90.

 Acceptability of IFA and MMS will be assessed by a quantitative questionnaire at the 30-day time point and the endpoint of the trial. This will allow us to capture participants’ opinions and perceptions of the products both relatively early in their use and after more extended exposure.

#### Organoleptic properties assessment

Participants will be asked a series of questions related to the organoleptic properties of the intervention products. These questions will cover various aspects of the products, including taste, smell, appearance, pill size, and color. To collect these data, participants will be presented with specific statements related to these properties, such as “I like the taste of the supplement” or “I like the color of the supplement.” Participants will then be asked to rate their level of agreement or disagreement with each statement using a Likert scale, which included response options like “strongly agree,” “agree,” “neutral,” “disagree,” and “strongly disagree.”

#### Quantitative analysis

To analyze and quantify the acceptability of IFA and MMS, we will use logistic regression. This statistical approach will allow us to compare the percentage of responses to each of the organoleptic questions for both IFA and MMS. By comparing participant responses, we assess the relative acceptability of the two intervention products based on various organoleptic properties.

### Interim analyses {21b}

No interim analyses are planned.

### Definition of analysis population relating to protocol non-adherence {20c}

Subjects who drop out of the study at an earlier stage will be tracked down for additional data collection via a home visit or phone survey. Every effort will be made to collect data until endpoint as long as the subject does not withdraw consent for further data collection. If necessary, missing data will be imputed. If there is missing data, assessment will be made if the data are missing completely at random (MCAR) or if the data are possibly missing due to observed or unobserved factors. Based on this assessment, imputation may or may not be required. If imputation of missing data is deemed necessary, we intend to employ appropriate imputation methods that align with the nature of the missing data and the assumptions underlying the imputation process.

#### We will conduct multiple imputation analyses for trial data

Multiple imputation is a robust approach that generates multiple imputed datasets, each with different imputed values based on observed data and specified models. It accounts for the uncertainty associated with imputation and can provide unbiased estimates and valid statistical inference. Lastly, if multiple imputation is conducted, we will perform sensitivity analyses to assess the robustness of our findings under different assumptions about the missing data mechanism and imputation methods. Sensitivity analyses allow us to evaluate the potential impact of missing data on our study results.

### Plans to give access to the full protocol, participant-level data and statistical code {31c}

Requests for access to the full protocol, participant-level data and statistical code will be considered by the corresponding author on a case-by-case basis, upon reasonable request and conditions.

## Oversight and monitoring

### Composition of the coordinating center and trial steering committee {5d}

The MMS Steering Committee members are responsible for providing technical review and inputs to the design of the study protocol; to review semi-annual progress reports; to help coordinate with relevant subnational stakeholders for smooth implementation of the clinical trial; observe study implementation and provide feedback and recommendations to the study team; provide technical interpretation of study findings; and help facilitate the sharing of project findings with relevant stakeholders.

### Composition of the data monitoring committee, its role and reporting structure {21a}

Written informed consent was obtained from each participant prior to any data collection activities, ensuring their voluntary participation and understanding of the study procedures. To oversee the trial and ensure its adherence to objectives, a Trial Steering Committee was established in Cambodia. The committee has various responsibilities, including reviewing and providing technical input on trial protocols and any amendments, monitoring the progress of the trial, overseeing the data safety monitoring processes, sharing relevant information, and addressing any issues that may arise during the trial. The committee played a crucial role in providing advice on trial design and offering guidance during the implementation phase.

### Adverse event reporting and harms {22}

All enrolled pregnant women will be provided with the contact information of the research project manager and the monitoring and evaluation manager on their copy of the informed consent form. Women will be asked to inform their health center midwife or doctor of any perceived adverse events related to the trial supplements.

### Frequency and plans for auditing trial conduct {23}

No plans for auditing trial conduct are expected.

### Plans for communicating important protocol amendments to relevant parties (e.g., trial participants, ethical committees) {25}

The established MMS Steering Committee (see “Composition of the data monitoring committee, its role and reporting structure {21a}”) will take the lead responsibility for sharing relevant information and addressing any issues that may arise during the trial.

### Dissemination plans {31a}

The results from this study will be shared with the MMS Steering Committee and the Scientific Advisory Board to inform an effective and feasible MMS scale-up strategy for Cambodia. Additionally, the findings will be shared globally with other stakeholders planning to scale up MMS in other locations. After all pregnant women enrolled in the adherence and acceptability trial have completed gestation and all analyses are completed, a national level dissemination will be held. Additionally, findings will be disseminated through several manuscripts published in peer-reviewed journal(s) and will be presented at academic conferences.

## Discussion

A limitation of the study design is the unblinded nature of our study design which has the potential to introduce bias that merit discussion. As participants in the trial will be informed of their assigned treatment arm, which includes receiving either IFA or MMS, this lack of blinding can introduce several forms of bias that need to be considered. First, there is the risk of performance bias, where healthcare personnel may inadvertently exert differential efforts to encourage adherence to MMS compared to IFA. To mitigate this risk, we have explicitly instructed all healthcare personnel and study team members to provide equal support and encouragement for both interventions. Participants are informed that both IFA and MMS provide essential nutrients necessary for maternal and fetal health and are encouraged to take their assigned supplements daily. Second, there is the possibility of participant bias. Since MMS is a new product in Cambodia, pregnant women and their influential family members may harbor hesitancy or concerns about this unfamiliar supplement, potentially leading to differential adherence rates between the two groups. IFA, on the other hand, has been widely promoted and is familiar to the population. To address this concern, our study includes an assessment of acceptability, aiming to understand the factors influencing pregnant women’s preferences and adherence to either IFA or MMS supplements.

Despite these limitations, our study design has many strengths to ensure bias was limited and processes in place to support rigorous data collection. Tablet counts will be conducted at various time points for all groups to monitor adherence accurately. Additionally, acceptability surveys with a Likert scale will be administered to assess participants’ organoleptic perceptions of the supplements.

Findings from this study will inform an effective and feasible MMS scale-up strategy for Cambodia. As it is already well established through a 2017 meta-analysis showing that supplementation with MMS provided greater reductions in the risk of low birthweight and preterm birth for infants born to undernourished and anemic women as compared to IFA. And, a 2019 Cochrane review compared the effects of MMS to IFA supplementation and found MMS was associated with reduced small for gestational age birth. If MMS adherence rates are non-inferior to IFA than the MMS Steering Committee plans to conduct a full costing analysis to assess if MMS can be procured at rates that are comparable to IFA and to assess other bottlenecks that may exist before undertaking a transition of their nutrition policy regarding micronutrient supplementation during pregnancy. Additionally, the findings will be shared globally with other stakeholders planning to scale up MMS in other locations.

## Trial status

The current protocol is Version 4 dated May 22, 2023. Ethical approval for the trial was obtained from the Cambodia MoH National Ethics Committee for Health Research (NECHR No 056) on September 1, 2022. The Trial was registered May 18, 2023, on ClinicalTrials.gov. Recruitment began March 1, 2023, and we anticipate recruitment to complete in July 2023.

### Supplementary Information

**Additional file 1: Appendix 1.** WHO Clinical Trials registry (Reviewer 1).

**Additional file 2: Appendix 2.** Informed Consent From.

## Abbreviations

ANC: Antenatal care
IFA: Iron folic acid
MMS: Multiple micronutrient supplementation
MoH: Ministry of Health
