# Supplementary material for: Adherence and acceptability of multiple micronutrient supplementation during pregnancy: Study protocol for a cluster-randomized non-inferiority trial in Cambodia
Source: Trials. 2024 Apr 29;25:289. doi: 10.1186/s13063-023-07891-z (PMC11057137; doi:10.1186/s13063-023-07891-z)
Supplement: Supplementary file 1 — Additional file 1: Appendix 1. WHO Clinical Trials registry (Reviewer 1). [file 13063_2023_7891_MOESM1_ESM.docx]

**Annex 1. WHO Clinical Trials registry**

| **WHO Trial Registration Dataset (Version 1.3.1)** | **Additional information** |
| --- | --- |
| **1. Primary Registry and Trial Identifying Number** | NCT05867836 (clinicaltrials.gov, registered May 18, 2023). |
| 2. Date of Registration in Primary Registry | NCT05867836 (clinicaltrials.gov, registered May 18, 2023). |
| 3. Secondary Identifying Numbers | N/A |
| 4. Source(s) of Monetary or Material Support | This study is funded through Vitamin Angels Alliance, a global non-profit organization with headquarters in Santa Barbara, California, USA. |
| 5. Primary Sponsor | The University of British Columbia  216-2205 East Mall  Vancouver BC \| V6T 1Z4 Canada  Phone +1 604 822 0421 |
| 6. Secondary Sponsor |  |
| 7. Contact for Public Queries | Corresponding author: Hou Kroeun, No 40, Street 348, Sangkat Toul Svay Prey 1, St 348, Phnom Penh, Cambodia, email: hkroeun@hki.org phone: +855 23 213 217 |
| 8. Contact for Scientific Queries | Crystal D. Karakochuk PhD, RD  The University of British Columbia  216-2205 East Mall  Vancouver BC \| V6T 1Z4 Canada  Phone +1 604 822 0421 |
| 9. Public Title | Adherence and acceptability of multiple micronutrient supplementation among pregnant women in Cambodia: A cluster-randomized non-inferiority trial |
| 10. Scientific Title | Adherence and acceptability of multiple micronutrient supplementation among pregnant women in Cambodia: A cluster-randomized non-inferiority trial |
| 11. Countries of recruitment | Cambodia |
| 12. Health Condition(s) or Problem(s) Studied | The study assesses the non-inferiority of acceptability and adherence rates of multiple micronutrient supplements (MMS) to iron and folic acid (IFA) supplementation during pregnancy. |
| 13. Intervention(s) | The trial will include three intervention groups.   1. MMS-180: Women enrolled in the MMS-180 group will receive 180 tablets of MMS distributed in one tablet bottle at their first antenatal care visit. 2. MMS-90: Women enrolled in the MMS-90 group will receive a total of 180 tablets of MMS (90 tablets will be distributed in one tablet bottle at their first antenatal care visit and another 90 tablets will be distributed at their second antenatal care visit). 3. IFA-90: Women enrolled in the IFA-90 arm will receive 90 tablets of iron folic acid (60 tablets in a bag at their first antenatal care visit and 30 tablets in a bag at their second antenatal care visit). |
| 14. Key Inclusion and Exclusion Criteria | Inclusion criteria:   - Aged 18-45 years - Gestational age at time of enrollment not greater than 14 weeks - Low risk pregnancy - Resides in Kampong Thom province, Cambodia, and is not planning to move in the next six months - Is not currently participating in any nutrition study, or are trying out a new governmental nutrition program that isn’t standard of care yet - Is willing to have data collectors visit the home every month |
| 15. Study Type | Randomized non-inferiority trial |
| 16. Date of first enrollment | March 1, 2023 |
| 17. Sample size | 1,545 pregnant women |
| 18. Recruitment Status | Complete: participants are no longer being recruited or enrolled |
| 19. Primary Outcome(s) | Primary Outcome: Non-inferiority of adherence rates (based on pill counts) to MMS-180 compared to IFA-90, using a non-inferiority margin of 15%. |
| 20. Key Secondary Outcomes | A secondary analysis will be to assess the non-inferiority of adherence rates of MMS-90 compared to IFA-90 as well as MMS-180 compared to MMS-90, all using a non-inferiority margin of 15%. |
| 21. Ethics Review | Cambodia MoH National Ethics Committee for Health Research (NECHR No 056) on September 1, 2022 |
| 22. Completion date | Trial ongoing. Anticipated completion date by December 31, 2023. |
| 23. IPD sharing statement | N/A for trials |
