# Supplementary material for: Adherence and acceptability of multiple micronutrient supplementation during pregnancy: Study protocol for a cluster-randomized non-inferiority trial in Cambodia
Source: Trials. 2024 Apr 29;25:289. doi: 10.1186/s13063-023-07891-z (PMC11057137; doi:10.1186/s13063-023-07891-z)
Supplement: Supplementary file 2 — Additional file 2: Appendix 2. Informed Consent From. [file 13063_2023_7891_MOESM2_ESM.docx]

**Annex 2. Informed Consent From**

Hello! My name is………………. I am here from Helen Keller Intl. Cambodia, an organization working for health, nutrition and agriculture.

We are conducting a research study **of people’s experience in the health system and asking questions that will help to improve some of the programs and policies for nutrition supplementation during pregnancy in Cambodia.** We are working together with The Ministry of Health in Cambodia and with an international organization called Vitamin Angels. We are trying to explore factors related to the acceptability and adherence to nutrition supplements by collecting data from pregnant women from three operations districts Staung, Baray-Santuk and Kampong Thom.

You have been selected for participation as you are pregnant and have enrolled in ANC visits in a health facility that is involved in the study. We are inviting you to be a participant in this study. We value your opinion and there are no wrong answers to our questions. We will use approximately an hour of your time. There will be no risk to you as a result of your participating in the study. Your participation in this study is completely voluntary. You are free to withdraw your consent and discontinue participation in this study at any time. All information gathered will be strictly treated as confidential and will be used only for the study purposes.

During this study, we will ask some questions related to your background and household, your current pregnancy, your access to health services and other details about the nutrition supplements (or nutrition ‘pills’) that you receive during your ANC visits. You will receive one of two different types of nutrition supplements in your ANC visit. Both supplements provide good nutrition for you and your baby and are safe to consume. We are interested about experiences of women who are taking these nutrition supplements – including how often you take them, your experience any side effects, or have any concerns related to the supplements. We will also ask to count the pill for IFA or weight the remaining pills for MMS every month from the supply that you received during your ANC visit.

If you have any questions or would like more information, you can contact either Rem Ngik (Monitoring and Evaluation Manager, Helen Keller Intl. Cambodia, email: [rngik@hki.org](mailto:rngik@hki.org) mobile: 078 95 16 12) or Meng Sokchea (Project Manager, Helen Keller Intl. email: msokchea@hki.org; mobile: 012 61 2 912).

You can say yes or no to participate. It is up to you to decide and there is no problem if you decide not to participate. Would you like to participate in the study? If so, please provide your name and signature below.

Name of person giving consent: ____________________________________

Signature of person giving consent: ____________________________________

Date of obtained consent: ____________________________________

Name of research staff obtaining consent: _____________________________
